# Sequential effects in preference decision: Prior preference assimilates current preference

**DOI:** 10.1371/journal.pone.0182442

**Published:** 2017-08-17

**Authors:** Seah Chang, Chai-Youn Kim, Yang Seok Cho

**Affiliations:** Department of Psychology, Korea University, Seoul, Korea; University of California, San Francisco, UNITED STATES

## Abstract

An important factor affecting preference formation is the context in which that preference decision takes place. The current research examined whether one’s preference formed for a previously presented stimulus influences the processing of a subsequent preference decision, henceforth referred to as the preference sequence effect. Using a novel sequential rating/judgment paradigm, the present study demonstrated the presence of a preference sequence effect using artistic photographs and face stimuli: A neutral stimulus was preferred more following a preferable stimulus than a less preferable stimulus. Furthermore, a similar trend was found even when the potential influence of response bias was controlled. These results suggest that an assimilative sequential effect exists even when sequential judgments are made solely based on one’s subjective feeling; preference formed for a preceding stimulus modulates preference for a subsequent stimulus. This implies the need for a consideration of trial sequence as a factor creating a psychological context affecting the subsequent preference decisions.

## Introduction

Imagine you are an artist and your work will be displayed in a prestigious art gallery. However, you found out that your work will be displayed next to a piece of work nominated as the most beloved artwork this year. Making things worse, a visitor path guides viewers through the gallery in a direction that guarantees the famous piece will be viewed just prior to your work. You are worried about any sequential influence the order of display may exert. It is possible that viewers do not like your work because preference for the preceding work was so high that preference for your work is contrasted against it. On the other hand, it is also possible that viewers like your work more because their preference for the preceding work assimilates current preference for your work.

A large body of evidence suggests that preference can be constructed and determined spontaneously in contexts. For example, the situational context in which a stimulus is placed influences a wide variety of preference decisions, including implicit racial preference [1], candidate preference decisions [2], musical preference [3], and aesthetic preference [4–6]. The influence of psychological context adds evidence to spontaneous construction and modulation of preference by contexts. An important piece of evidence comes from affective priming studies in which a prior exposure to an emotional stimulus influences subjective judgments of a neutral stimulus. Priming research has shown the influence of affective primes on subsequent subjective judgments [7–9]. When people were briefly presented with a positive or negative emotional stimulus prior to a neutral target stimulus, preference for the target was modulated by the valence of the preceding emotional stimulus [7–12]. Furthermore, the stronger effects of unconscious affective priming compared to conscious one have been found and attributed to the diffuse quality of affective reaction in which unconscious affect can “spill over” onto unrelated stimuli [8, 13]. This kind of misattribution of affect has also been found in the *affect misattribution procedure (AMP)*. In the AMP, participants are asked to perform pleasantness judgments for neutral targets while ignoring a briefly preceding affect-related picture prime [14]. Even though people intend to ignore the affect-laden stimulus, neutral targets are more positively evaluated following positive picture primes than following negative picture primes.

Even though priming research revealed the influence of a preceding emotional stimulus on a current neutral stimulus, they used different types of stimuli for primes and targets, and responses were made only for the targets but not for the primes. In other lines of research, in contrast, sequential effects of subjective judgments to sequentially presented stimuli were examined. With a variety of stimulus types, it has been repeatedly shown that responses to current stimuli were significantly influenced by a preceding stimulus when people sequentially evaluated facial expressions [15], facial attractiveness [16–19], sports performance [20–21], essay ratings [22], and prices [23]. Rich evidence suggests that sequential context or trial-by-trial transition plays a pivotal role in shaping behavioral responses in a variety of contexts and comparison processes happen in a relatively spontaneous, effortless, and unintentional manner [20, 24].

Two types of effects have been observed in sequential contexts: Assimilation and contrast effects. The *assimilation effect* refers to a positive relationship between judged values of a target stimulus and a contextual stimulus accompanying the target whereas the *contrast effect* refers to a negative relation between these two values [25]. Since the terms assimilation and contrast refer to the direction of context effects rather than to the specific underlying processes [26], different kinds of assimilation and contrast effects can be generated based on different kinds of processes. Mussweiler (2003) proposed a selective accessibility model of comparison consequences in which the perceived similarity between the standard and target determine assimilative versus contrastive comparisons [27]. According to this model, a focus on similarities leads to assimilation effects whereas a focus on dissimilarities leads to contrast effects [20]. On the other hand, studies on affective reactions have shown that behavior tends to be in opposite directions depending on participants’ awareness of a target stimulus or the relationship between the context and the target stimulus [25, 28–32]. When participants are unaware of an influencing stimulus or the association between an affective stimulus and a neutral target, they tend to elicit an assimilation effect reacting in accordance with the attributes of the influence. However, when participants are aware of the influence of a prime, a contrast effect tends to be produced [33]. Additionally, the availability of additional cognitive resources and efforts to resist the initial reaction to the prime has been suggested as another determinant for assimilation or contrast effects [25, 31, 34–35]. The contrast effect is often accompanied by more detailed processing with larger contribution of cognition for affective information, which dovetails with a traditional view that the longer an affective stimulus is presented, the more information about the stimulus is gained [36].

Judgments tested by previous studies seem to have some types of standard or absolute criterion a person can compare to besides their subjective feeling. In such situations, participants may have some accessible knowledge which they can use for judgments. For example, in sports judgment situations, they are likely to have access to a knowledge related to the performance evaluation such as speed, and this knowledge functions as a standard to compare with previous players. Also, in attractiveness judgments, people are likely to think about a standard of beauty in a certain culture, and this may lead them to easily access relevant knowledge influencing the subsequent comparison process. However, when a judgment should be made solely based on one’s subjective feeling, do sequential effects still exist? When the only relevant standard is their preferential feeling itself, does this also elicit sequential effects? When people make sequential preference decisions for a current stimulus in experimental or practical settings, one’s preference formed in a preceding trial may form a psychological context automatically, anchoring and biasing a subsequent decision and eliciting a *preference sequence effect* in a trial-by-trial manner. A preference-related valence state formed in the preceding trial may linger on and influence the processing of a subsequent preference.

### The present study

The present study aimed to explore whether prior preference itself is enough to form a psychological context influencing a subsequent preference decision. To test for the presence of the preference sequence effect, artistic photographs and face stimuli were used as experimental stimuli, based on the following rationale. First, since the situations where preference formation is critical such as museums, exhibitions, or advertisements usually involve a sequential presentation of stimuli or events, the sequential modulation of preference in a stimulus set used in many practical settings, if any, should be carefully considered. Second, faces are one of the most frequently used stimuli in preference studies which test preference-related mechanisms through a sequential presentation of stimuli. As a randomly determined sequence might include a preferable stimulus preceding a neutral stimulus or a less preferable stimulus preceding a neutral stimulus, the preference sequence effect in face stimuli is worth testing.

In the present study, we manipulated the sequence of preferable/less preferable stimulus and neutral stimulus, and tested whether preference to a current neutral stimulus changes depending on preference for a preceding preferable/less preferable stimulus. Experimental stimuli were carefully selected through a preliminary preference rating procedure prior to the main experiments. In the preliminary procedure, a group of participants completed preference ratings for a number of randomly presented artistic photographs and another group performed the identical procedure for faces. Based on the preliminary rating data, experimental stimuli were chosen and classified into preferable/less preferable stimuli and neutral stimuli. The selected artistic photos were used for Experiments 1, 3, and 4, whereas the selected face stimuli were used for Experiment 2. In the main experiments, four newly recruited groups of participants performed tasks. In a general procedure, a preferable or less preferable stimulus and then a neutral stimulus were sequentially presented in a pairwise manner and the influence of the preceding stimulus on the subsequent preference rating of the current stimulus was examined. Since previous studies revealed that the stimulus right before the target (N-1 or 1-back stimulus) serves as the standard for comparison [20] or influences subjective judgments for a target [16], pairing two items should be sufficient to test the sequential effects. Damisch et al. (2006)’s study 2 also used a similar paradigm in which a pre-rated moderate distance jump followed either a pre-rated long or short distance jump, and the influence of previous rating for a long or a short image on rating for the moderate image was measured [20]. However, in their experiment, only three pictures were used and the effects of previous image were tested in a between-subject design. On the other hand, in the current design, the stimulus set included 30 preferable, 30 less preferable, and 30 neutral stimuli which were all tested in each participant, allowing within-subject contrast for the effects of a preceding preferable and less preferable stimulus.

It was hypothesized that if people adjust their preference to the preference formed in a prior judgment, preference for a neutral stimulus would differ depending on the preferable or less preferable nature of the preceding stimulus. On the other hand, if the preceding preference does not influence the formation of preference for a current stimulus, preference for a neutral stimulus will remain constant regardless of whether the preceding stimulus was preferable or less preferable. The present study will provide empirical evidence for whether one’s preference itself for a preceding stimulus influences the preference evaluation of a subsequent stimulus. This study also bears theoretical implications by adding evidence for what conditions should be met for sequential bias in preference decisions, which will further provide practical considerations.

## Experiment 1

The aim of Experiment 1 was to investigate whether a preference formed for a preceding artistic photograph influences the subsequent preference judgment. If the preceding preference affects the subsequent preference, preference ratings for the current stimulus would differ depending on the preceding preference induced by the previously seen and judged stimulus. To examine this possibility, the sequence of preferable/less preferable stimulus and neutral stimulus was manipulated.

Based on the results from the preliminary preference rating, preferable and less preferable stimuli and neutral stimuli were selected. In the main experiment, participants were asked to perform a preference rating task for pairs of artistic photographs which were sequentially presented in a trial. While participants were not aware of the sequence manipulation, a preferable or less preferable photo was presented first and followed by a neutral photo. Each neutral stimulus was presented twice by following a preferable stimulus in one block and a less preferable one in another block, respectively, in order to prevent possible confounds arising from the variability of stimuli and to test the change of preference within each stimulus. Since repetition of prime-like stimuli diminishes their effectiveness [9], a preferable or less preferable stimulus was never repeated. Sequence effects were measured based on changes in preference ratings for neutral stimuli that followed the presentations of either preferable or less preferable stimuli. If a sequence effect exists, preference ratings for the same neutral stimulus would differ following a preferable or less preferable stimulus.

### Preliminary preference rating

#### Participants

Twelve undergraduate and graduate students (mean age = 24.67, 9 females) at Korea University participated in the experiment for payment of KRW 3,000 (about 3 US dollars). All participants had normal or corrected-to-normal vision, and had no professional knowledge of artistic photographs based on the self-report. The present and following experiments were approved by the institutional review board at Korea University (KU-IRB-13-28-A-1), and a written informed consent was obtained from all participants prior to the testing.

#### Materials and apparatus

150 images of artistic photographs were collected from the Internet. The photos were chosen mainly to induce more variety of preference. They typically depicted scenery but some of them contained several people. Photos portraying a specific person were not chosen because the person could affect preference instead of overall impression of a photo. Stimuli were resized to fit within a frame of 450 x 330 pixels and the background color was white (79.81 cd/m^2^). Stimuli were presented randomly but in a way that a preferable or less preferable stimulus always preceded a neutral stimulus. Each stimulus remained on the screen until a response was recorded. The experiment was programmed and presented using E-Prime software (Version 2.0, Psychology Software Tools, Inc.). Stimuli were presented on a 17-inch CRT monitor of a personal computer and viewed at a distance of approximately 60 cm from the participants. Keyboard responses consisted of ‘z’, ‘x’, ‘c’, ‘v’, ‘b’, ‘n’, ‘m’ keys labeled ‘-3’, ‘-2’, ‘-1’, ‘0’, ‘1’, ‘2’, ‘3’, respectively. The numbers indicating rating scores were also displayed at the bottom of the screen.

#### Procedure

The experiment took place in a soundproof booth. Participants were instructed to rate an assortment of photos on a scale of -3 to 3 based on their immediate preference with -3 indicating “do not like at all”, 0 indicating “neither like nor dislike”, and 3 indicating “like very much.” The 150 photographic stimuli were presented one after another with a rating recorded after each image. The preliminary rating procedure lasted for 10 minutes.

#### Stimulus selection

The data from the preliminary preference rating were transformed into normalized scores (Z scores); the individual mean scores and standard deviations for each participant were calculated and the value calculated by subtracting the mean from each rating point was divided by the standard deviation. With those Z scores, the large individual difference in preference rating (each participant had a mean rating score ranging from—0.55 to 1.69) was controlled and the group mean scores could be computed for each image. Images were arranged in order of their mean scores across participants and selected based on the scores. The mean Z scores of all images ranged from -1.99 to 1.09, and the standard deviation of the mean Z scores across images was 0.65. The 30 images with mean Z scores above 0.25 were selected as preferable stimuli and 30 images with mean Z scores below -0.25 were selected as less preferable stimuli. In addition, 30 images with mean Z scores between -0.2 and 0.2 were selected as neutral stimuli. The images with a small standard deviation were primarily selected over the ones with a larger standard deviation. The mean Z scores and standard deviations of the images selected as less preferable, preferable, and neutral ones are listed in Table 1.

**Table 1:** The mean Z scores and SD for all selected images for Experiments 1, 3, and 4.

	Preferable	Neutral	Less preferable
Mean	0.53	0	-0.91
SD	0.20	0.1	0.46

### Main experiment

#### Participants

Sixteen new students (mean age = 25.38, 8 females) at Korea University participated in the experiment for payment of KRW 3,000 (about 3 US dollars). All participants had normal or corrected-to-normal vision, and had no professional knowledge of artistic photographs based on self-report.

#### Materials and apparatus

The materials and apparatus were identical to the preliminary rating procedure, except as noted. The 90 photographic images selected through the preliminary rating procedure, including 30 preferable, 30 less preferable, and 30 neutral ones, were used as experimental materials.

#### Design and procedure

Given the wide variety of complexity and features in artistic photos, a paired sequence design was employed, which allows for comparing the effects of prior stimulus for the same current stimulus within the same participant. A paired sequence design involves two stimuli in each trial. The first stimulus was from either the preferable or less preferable set evaluated during the preliminary rating procedure. The second stimulus was from the group of stimuli which were previously judged as neutral. In this design, the same neutral stimulus follows a preferable stimulus once and a less preferable stimulus once in two separate blocks. As the nature of object associated with affect influences the impact of affective reactions [37], this manipulation enables a “within-target stimulus” statistical test avoiding the influence of intrinsic preference differences among neutral stimuli. Each block consisted of the presentations of 15 preferable stimuli preceding 15 neutral stimuli and 15 less preferable stimuli preceding 15 neutral stimuli. Each participant was presented with 30 pairs of randomly presented stimuli per block. In the second block, the 15 neutral stimuli which had been paired with preferable stimuli in the first block were paired with less preferable stimuli and vice versa. Preferable or less preferable stimuli were never repeated throughout the experiment.

**Fig 1:**
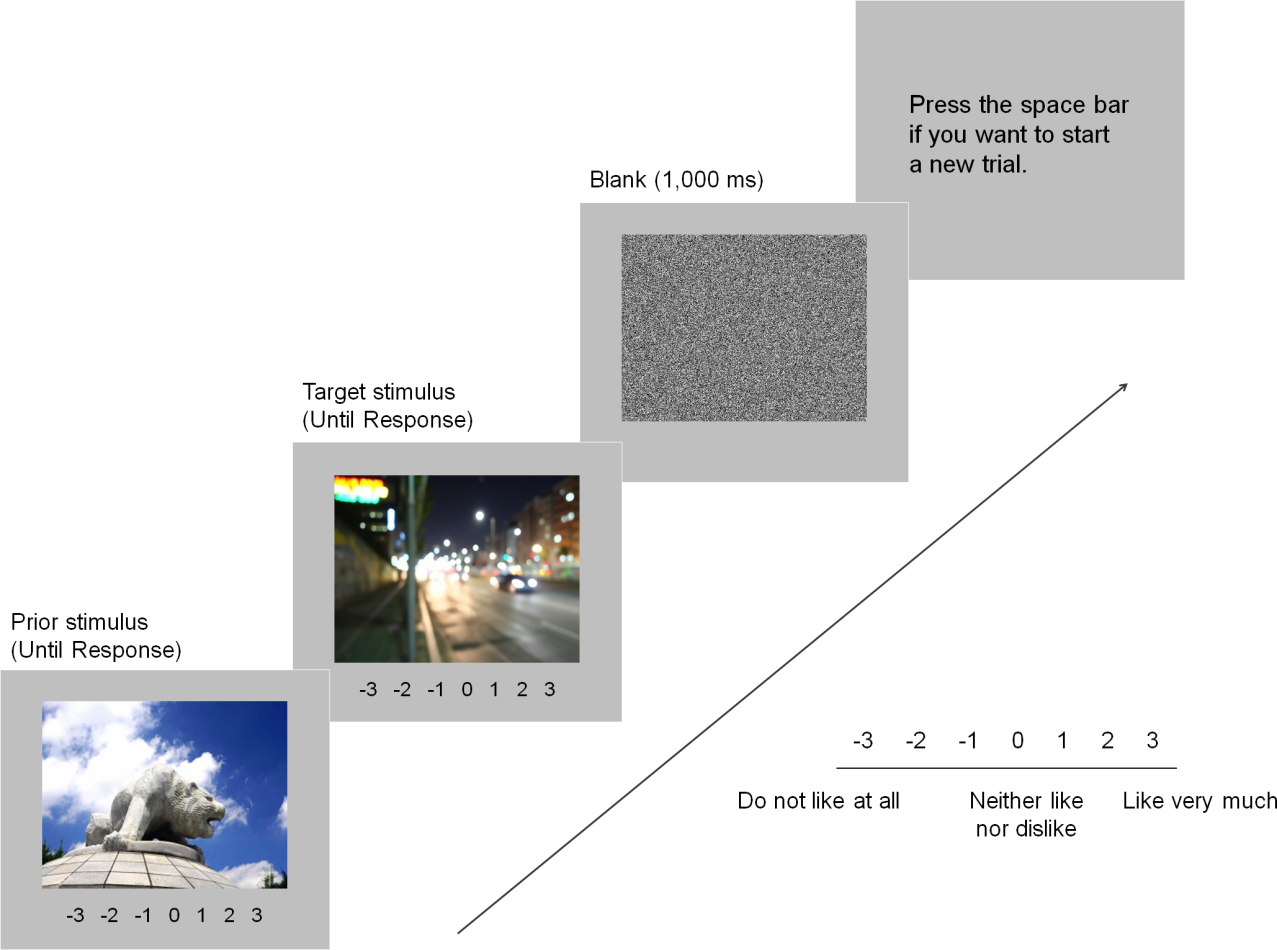
Example of a trial sequence in Experiment 1. Participants were instructed to rate a presented stimulus based on their immediate preference. A preferable or less preferable stimulus was presented first and a neutral stimulus followed it. After responses were recorded for two consecutive stimuli, a blank screen appeared for 1,000 ms and participants were instructed to press the spacebar to start the next trial.

Participants were asked to rate each presented stimulus on a scale of -3 to 3 based on their immediate preference with -3 indicating “do not like at all”, 0 indicating “neither like nor dislike”, and 3 indicating “like very much.” The numbers indicating ratings were also displayed at the bottom of the screen. Even though responses were neither timed nor speeded, participants were encouraged not to ponder on it but to respond following an initial impression. Participants were also instructed to place their seven fingers (three left fingers and four right fingers) on the keyboard row of number-indicating keys so that ratings could be made easily and quickly. Each stimulus remained on the screen until a response was recorded, and after rating two stimuli sequentially in a trial, participants were instructed to press the spacebar in order to begin the next trial (see Fig 1). It was also emphasized that some of the images which were shown once might be presented again but they should rate them based on the immediate preference at the moment, rather than remembering the previous ratings on them. The experiment lasted for about 10 minutes.

#### Debriefing

After finishing the experiment, participants were asked to guess the purpose of the experiment and they were debriefed. None of them predicted the purpose correctly and participants reported that they were never aware of this sequence manipulation.

### Results and discussion

Stimulus ratings for each participant were transformed to Z scores for statistical analyses. As expected, the responses to preferable or less preferable stimuli were different, validating the initial classification of artistic photos according to preference: Rating responses to preferable and less preferable stimuli were significantly different both in block 1, *t*(15) = 13.09, *p* < .0001, *d* = 1.88, and in block 2, *t*(15) = 10.95, *p* < .0001, *d* = 1.35. The mean Z scores and the standard deviations of preferable and less preferable stimuli in the two blocks are listed in Table 2.

**Table 2:** The mean Z scores (SD) for preferable and less preferable stimuli in Experiment 1.

	Preferable	Less preferable
Block 1	0.70 (0.68)	-0.80 (0.90)
Block 2	0.55 (0.62)	-0.45 (0.84)

Based on the obtained validity of preferable/less preferable stimuli, the mean responses for current stimuli were calculated for each participant as a function of prior stimulus preference, and a repeated-measure analysis of variance (ANOVA) was conducted on the mean response data for current stimuli with prior stimulus preference as a within-participant factor. According to the results from the analysis, the main effect of prior stimulus preference was significant, *F*(1, 15) = 16.835, *p* = .001, $\eta_{p}^{2}$ = .529. The mean current preference rating when the prior stimulus was a preferable one (*M* = .0718, *SEM* = 0.0174) was higher than when that was a less preferable one (*M* = -.0720, *SEM* = 0.0177). The results suggest that the preference ratings for the same neutral stimulus changed depending on the preference for the preceding stimulus. A neutral stimulus was rated more preferably when it followed a preferable stimulus than a less preferable one.

It should be noted that two stimuli were presented as independent stimuli that should be judged respectively while participants were unaware of the critical manipulation. In other words, participants were consciously aware of a prior stimulus as an independent stimulus which has nothing to do with the processing of the subsequent stimulus and the preference rating for the prior stimulus was answered before the subsequent neutral stimulus was presented. Nonetheless, the preference ratings for a neutral stimulus were different as a function of the preference for a preceding stimulus. This demonstrates that, in a sequence of stimulus presentation, the preceding stimulus event can act as an affective context influencing the preference judgment for following stimulus.

Though a sequence effect was observed with artistic photograph stimuli in Experiment 1, they do not comprise a commonly used type of stimuli in preference studies. Therefore, in a separate experiment, we tested whether the obtained sequence effect can be generalized to another, more frequently used type of stimuli.

## Experiment 2

Experiment 2 examined whether the preference sequence effect found using artistic photographs in Experiment 1 is also present when participants make preference decisions for human faces. Face stimuli were widely used in preference studies because faces are a familiar stimulus about which we make preference decisions frequently in real life [38] and faces elicit immediate innate affective reactions [39–40]. Faces have also been known to be subject to a low-level perceptual adaptation effect [41–42]. Using female faces with neutral expression, Rhodes et al. (2003) found that brief exposure to configurally distorted faces altered which face look most attractive by adapting distortion. Following adaptation to compressed faces, preference toward undistorted average faces was reversed.

Just as in Experiment 1, the preliminary preference rating for face stimuli was performed prior to Experiment 2. The procedure was identical to that of Experiment 1 except that the stimuli were female faces whose visual features were controlled. We adopted only female faces as experimental stimuli to minimize sex differences of face stimuli in preference decisions [17, 43–46]. Even though some previous work has presented both male and female faces in a randomly ordered sequence and tested sequential effects [16], other studies found effects of sex on sequential effects [17, 46]. Preferences for female faces, compared to male faces, were also known to be less susceptible to contextual information [10].

In the main experiment, participants performed a preference rating task for the presented face stimuli. While participants were not aware of the sequence manipulation, a preferable or less preferable face always preceded a neutral face. As in Experiment 1, sequence effects were measured based on changes in preference ratings for neutral stimuli that followed the presentations of either preferable or less preferable stimuli. The presence of a sequence effect would reveal that face stimuli are also subject to the sequential modulation of the preceding stimulus.

### Preliminary preference rating

#### Participants

Twelve students (mean age = 24, 6 females) at Korea University participated for payment of KRW 3,000 (about 3 US dollars). All participants had normal or corrected-to-normal vision based on the self-report.

#### Materials and apparatus

150 female face images were taken from our in-house face database and various Internet websites, and were all in frontal view. All the faces were similar ages (in their twenties) and showed neutral facial expression. All of them were Asian faces (Koreans). Stimuli were resized to fit within a 120 x 160 pixels frame and the locations of eyes and sizes of faces were matched approximately. To minimize the influence of other visual factors including color and contrast, all the stimuli were converted to greyscale and the background was white (79.81 cd/m^2^). The RMS contrasts were calculated and equalized by using Matlab software (R2011a, MathWorks) and Psychophysics toolbox (Ver. 3, Psychophysics Toolbox, [47–48]). The experiment was programmed and presented using E-Prime software (Version 2.0, Psychology Software Tools, Inc.). Stimuli were presented on a 17-inch CRT monitor of a personal computer and viewed at a distance of approximately 60 cm. Keyboard responses consisted of ‘z’, ‘x’, ‘c’, ‘v’, ‘b’, ‘n’, ‘m’ keys labeled ‘-3’, ‘-2’, ‘-1’, ‘0’, ‘1’, ‘2’, ‘3’, respectively.

#### Procedure

Participants were instructed to rate an assortment of face images on a scale of -3 to 3 based on their immediate preference with -3 indicating “do not like at all”, 0 indicating “neither like nor dislike”, and 3 indicating “like very much.” Images remained on the screen until a response was recorded. The preliminary rating procedure lasted for 10 min.

#### Stimulus selection

The data from the preliminary rating procedure were transformed to Z scores, and the mean scores across participants were calculated for each image. Images were listed in order of their mean scores across participants and selected based on the mean scores. The calculated mean Z scores of all images ranged from -1.28 to 1.65, and the standard deviation of the mean Z scores across images was 0.65. 30 images whose mean Z scores were above 0.5 were selected as preferable stimuli and 30 images whose mean Z scores were below -0.5 were selected as less preferable stimuli. In addition, 30 images whose mean Z scores were between -0.2 and 0.2 were selected as neutral stimuli. The images with a small standard deviation were primarily selected over the ones with a larger standard deviation. The mean Z scores and standard deviations of the selected images as preferable, less preferable, and neutral ones are shown in Table 3.

**Table 3:** The mean Z scores and SD for all selected images for Experiment 2.

	Preferable			Neutral	Less preferable
Mean		0.92	0		-0.84
SD	0.29			0.12	0.19

### Main experiment

#### Participants

Sixteen new students (mean age = 24.94, 8 females) at Korea University participated for payment of KRW 3,000 (about 3 US dollars). All participants had normal or corrected-to-normal vision based on the self-report.

#### Materials and apparatus

The materials and apparatus were identical to the preliminary preference rating procedure, except as noted. The 90 stimuli selected through the preliminary rating procedure were used as experimental materials. These included 30 preferable, 30 less preferable, and 30 neutral ones.

#### Design and procedure

The design and procedure were identical to Experiment 1, except the stimuli. Face stimuli consisting of less preferable, preferable, and neutral female face images were used as preceding or current stimuli, instead of artistic photograph stimuli.

#### Debriefing

After finishing the experiment, participants were asked to guess the purpose of the experiment and they were debriefed. None of them predicted the purpose correctly and participants reported that they were never aware of this sequence manipulation.

### Results and discussion

Stimulus ratings for each participant were transformed to Z scores for statistical analyses. As expected, the responses to preferable/less preferable stimuli were different, which verifies the initial classification of faces according to preference: Rating responses to preferable and less preferable stimuli were significantly different both in block 1, *t*(15) = 11.92, *p* < .0001, *d* = 1.66, and in block 2, *t*(15) = 11.62, *p* < .0001, *d* = 1.70. The mean Z scores and the standard deviations of preferable and less preferable stimuli in the two blocks are listed in Table 4.

**Table 4:** The mean Z scores (SD) for preferable and less preferable stimuli in Experiment 2.

	Preferable	Less preferable
Block 1	0.71 (0.79)	-0.62 (0.81)
Block 2	0.57 (0.81)	-0.67 (0.64)

Based on the obtained validity of preferable/less preferable stimuli, the mean responses for neutral stimuli were calculated for each participant as a function of prior stimulus preference and a repeated measure ANOVA was conducted on the mean response data for neutral stimuli with prior stimulus preference as a within-participant factor. According to the results from the analysis, the main effect of prior stimulus preference was significant, *F*(1, 15) = 10.524, *p* = .005, $\eta_{p}^{2}$ = .412. The mean target preference rating when the prior stimulus was a preferable one (*M* = .1135, *SEM* = 0.0348) was higher than when the prior stimulus was a less preferable one (*M* = —.1125, *SEM* = 0.0349). The results suggest that the preference for the same neutral face stimulus changed depending on the preference for the preceding stimulus.

A significant sequence effect was found with a more general type of stimulus, i.e., face, in Experiment 2. As in Experiment 1, the obtained sequence effect indicates that preference for a previously seen and judged face can influence preference for a currently presented face. People adapted their preference to previously formed preference: With face stimuli, preference reversals were found by high level features (preferences) as well as low level features (distortion; [42]).

Experiments 1 and 2 revealed the preference sequence effect with a preference rating procedure. However, even though a rating task is sensitive enough to capture the extent to which the preference changes, preference is often judged in a binary way: whether it is preferred or not. Therefore, further research was required to test whether the preference sequence effect we observed with the rating task can be generalized under a binary decision-making situation.

## Experiment 3

The aim of Experiment 3 was to examine whether the sequence effect is replicated with a binary choice task procedure. The evidence of the preference sequence effect found in Experiments 1 and 2 was obtained by calculating the changes of rating value for the same neutral stimulus depending on prior stimulus preference. However, it is common in the preference literature for a binary choice (prefer or not) procedure to be employed to investigate the underlying mechanism of preference [38]. The binary choice task has been widely adopted due to its advantages producing no memory load and minimal response bias effects [49]. Since preference is a component underlying people’s evaluations of objects and subsequent decision making such as purchase of an object or selection of a person, replication of the obtained finding with a binary choice task would have more ecological implications. Taubert et al. (2016) also used a binary attractiveness task to examine sequential effects in attractive judgment in an online dating situation [19].

In Experiment 3, participants were asked to respond whether they prefer or do not prefer the presented artistic photograph. The sequence effect was measured based on the percentage of preferred or non-preferred responses, following presentations of either preferable or less preferable stimuli.

### Materials and methods

#### Participants

Sixteen newly recruited undergraduate and graduate students (mean age = 23.31, 9 females) at Korea University participated for payment of KRW 3,000 (about 3 US dollars). All participants had normal or corrected-to-normal vision, and had no professional knowledge of artistic photographs based on the self-report.

#### Materials, apparatus, and procedure

The materials and apparatus were identical to Experiment 1, except as noted. Participants performed a preference judgment task in which they were instructed to press the left or right key to indicate whether they prefer or do not prefer the presented photo stimulus. The mapping was counterbalanced across participants. Since left-right judgments are simple and do not require searching the appropriate response button from several options, stimuli were presented in a limited duration: a preferable/less preferable stimulus was presented for 2,500 ms, followed by a neutral stimulus also shown for 2,500 ms. Participants were asked to respond to each stimulus as quickly as possible while the stimulus is displayed.

#### Debriefing

After finishing the experiment, participants were asked to guess the purpose of the experiment and they were debriefed. None of them predicted the purpose correctly and participants reported that they were never aware of this sequence manipulation.

### Results and discussion

A repeated-measure ANOVA was conducted on the proportions of responses with prior stimulus preference and current stimulus preference as within-participant variables. As stimuli were presented for a limited duration, additional ANOVAs were conducted on the mean RTs for the preferable/less preferable stimulus with the prior stimulus preference as a within-subject variable and on the mean RTs for the neutral stimulus with the prior stimulus preference and neutral stimulus response as within-subject variables.

#### Proportions of responses as a function of prior and current stimulus preferences

**Fig 2:**
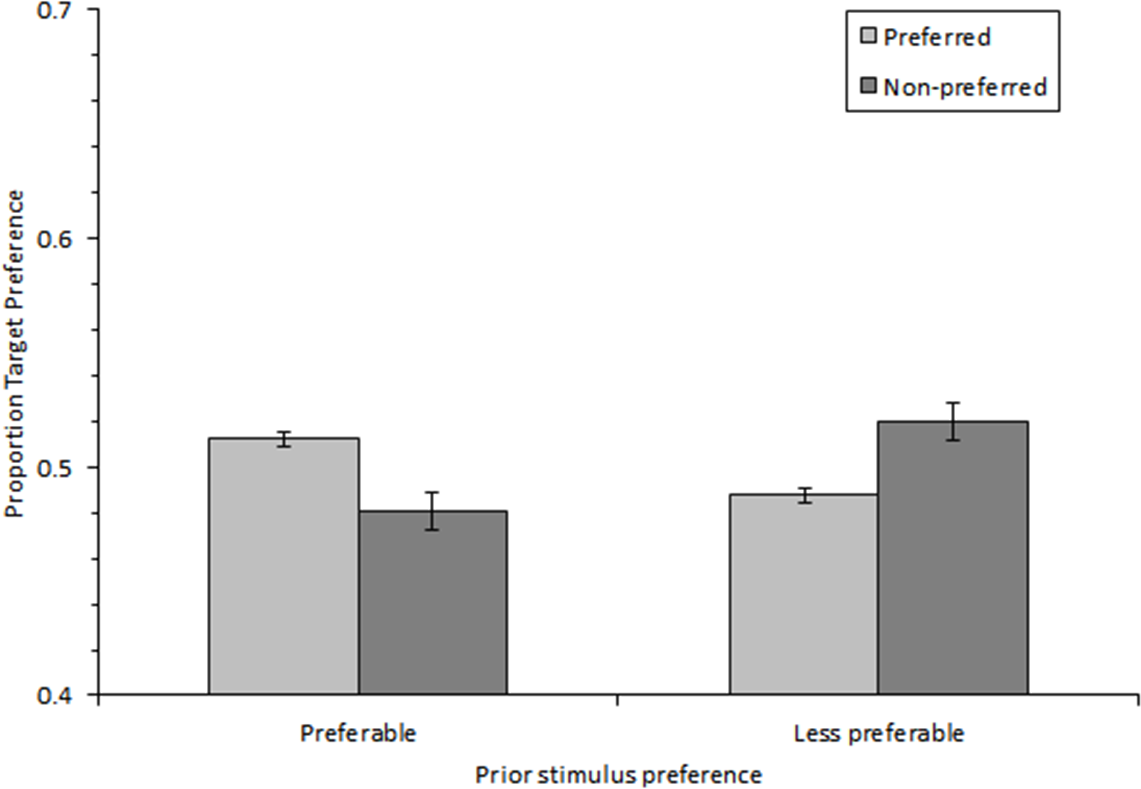
The proportions of responses as a function of prior and current stimulus preferences in Experiment 3. The percentages of the preferred and non-preferred responses were significantly different depending on the prior stimulus preference (*p* = .008). After a preferable stimulus, the percentage of preferred responses to the following neutral stimulus was higher than that of non-preferred responses whereas the opposite pattern was observed after less preferable stimulus. Error bars represent standard error of the mean.

The main effect of current stimulus preference was significant, *F*(1, 15) = 5.787, *p* = .029, $\eta_{p}^{2}$ = .278. The main effect of prior stimulus preference was not significant, *F*(1, 15) = 1. The interaction between prior preference and current preference was significant, *F*(1, 15) = 9.340, *p* = .008, $\eta_{p}^{2}$ = 0.384. The percentage of preferred response for the neutral stimuli was higher when following preferable stimuli (*M* = 51%) than less preferable ones (*M* = 49%). The percentage of non-preferred response for the neutral stimuli was higher when following less preferable stimuli (*M* = 52%) than preferable ones (*M* = 48%; see Fig 2).

#### RTs for preferable/less preferable stimuli

The main effect of prior stimulus preference was significant, *F*(1, 15) = 17.186, *p* = .001, $\eta_{p}^{2}$ = .534. Faster RTs were obtained with preferable stimuli (*M* = 930 ms) than less preferable stimuli (*M* = 1,080 ms).

#### RTs for neutral stimuli

The main effect of neutral stimulus response was significant, *F*(1, 15) = 11.116, *p* = .005, $\eta_{p}^{2}$ = .426. Preferred responses (*M* = 905 ms) were faster than non-preferred responses (*M* = 1,056 ms), which is consistent with the tendency of RTs for preferable/less preferable stimuli. The main effect of prior stimulus preference did not reach significance, *F*(1, 15) < 1, indicating that preceding stimulus did not influence the subsequent reaction time for current neutral stimuli. The interaction between prior stimulus preference and current neutral stimulus response was also not significant, *F*(1, 15) < 1. The absence of interaction demonstrates that reaction time for neutral stimuli did not differ following preferable and less preferable stimuli.

When a binary choice task was adopted, we observed results consistent with those of Experiments 1 and 2. In Experiment 3, observers made a higher percentage of “prefer” judgments for neutral stimuli when a preferable stimulus preceded them than a less preferable one did. This indicates that people tend to prefer the present stimulus after viewing a preferable stimulus than a less preferable stimulus, whereas the opposite is true when the preceding stimulus was a less preferable one. The sequential influence came into play, even when people make binary choice decisions for their preference toward artistic photograph stimuli.

However, RT data did not reveal any significant interaction regarding the preference sequence effect, even though significantly faster responses were observed for prior preferable stimuli and preferred neutral stimuli, respectively. The faster responses obtained for preferable stimuli than less preferable stimuli are consistent with previous findings that the preference judgment time varied as a function of preference [50–51]. These results might indicate more efficient processing of preferable stimuli than less preferable ones. However, the lack of the interaction between prior preference and current response implies that prior stimulus preference did not modulate the processing efficiency of the subsequent neutral stimulus.

Even though consistent results were found across three experiments, it should be noted that there is a possibility that a response bias had affected the obtained results. One might argue that the obtained sequence effects result not from sequence effects of preference *per se*, but rather from sequence effect in response biases independent of preference. In other words, people might have made “prefer” responses more after making a “prefer” decision for the previous stimulus than after making a “non-prefer” decision for it, because they simply wanted to stay around the response point they just made. In order to test this potential interpretation of the results from Experiments 1–3, Experiment 4 was conducted.

## Experiment 4

The purpose of Experiment 4 was to examine the response bias hypothesis and minimize the sustained visual influence of the preceding stimulus. It is possible that participants were simply not willing to move their responding hand placed on a response key toward the other key to change their response. That is, the tendency to stay in the same place might have influenced the results from Experiments 1, 2, and 3. Also, the visual afterimage of the prior stimulus might have remained and influenced subsequent processing of the neutral stimulus. Experiment 4 examined these possibilities where the preferable/less preferable stimulus was just passively viewed by participants without any explicit preference judgments and a mask display was inserted between the preceding and current stimuli to remove a sustained visual effect of the prior stimulus. Participants might not even have a mindset for judging their preference for a preferable/less preferable stimulus because participants were told to detect whether a red dot appears in the middle of the stimulus. Participants performed a dual task in which a dot detection task and a preference rating task were combined. During the dot detection task participants were asked to fixate on the center of the stimulus to detect a red dot, which enabled implicit processing of the stimulus while a red dot was absent.

### Materials and methods

#### Participants

Twenty four new students (mean age = 23.25, 12 females) at Korea University participated for payment of KRW 3,000 (about 3 US dollars). We increased the number of participants to twenty four because weaker sequence effects were expected since participants did not respond to a prior preferable/less preferable stimulus in this design. All participants had normal or corrected-to-normal vision, and had no professional knowledge of artistic photographs based on the self-report.

#### Materials, apparatus, and procedure

**Fig 3:**
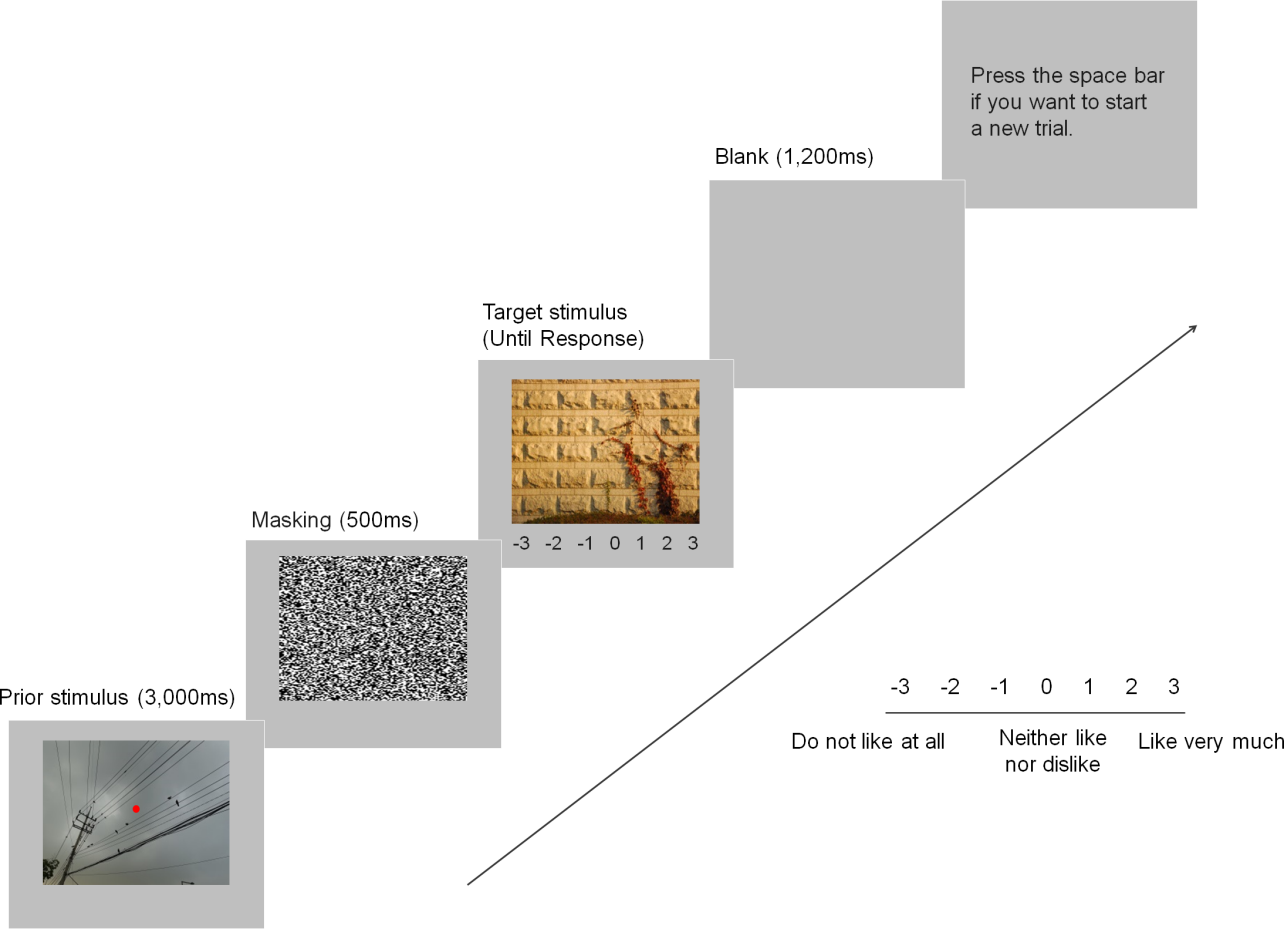
Example of a trial sequence in Experiment 4. A prior stimulus which did not require any response was presented for 3,000 ms, while filler stimuli which were not included in the analysis required a response. After a mask (500 ms) following a preferable/less preferable stimulus, a neutral stimulus was introduced and remained on the screen until a response was made. After a response was recorded, a blank screen appeared for 1,200 ms and participants were instructed to press the spacebar to start the next trial.

The materials and apparatus were identical to Experiment 1, except as noted. Participants performed a dot detection task and preference rating task sequentially with use of different hands in each trial. While a preceding stimulus was presented for 3,000 ms, participants were asked to press the designated button “z” with their left index finger if they detect a red dot in the middle of the stimulus, or they were supposed to look at it until it disappeared. The stimuli including a red dot and the subsequently presented paired stimuli were fillers which accounted for one third of trials. Those additional stimuli were chosen randomly from the previously obtained stimulus set based on the preliminary preference rating procedure, which had not been used for the main experiments. Filler stimuli were never repeated during the experiment. The preferable/less preferable stimulus was followed by a mask which lasted for 500 ms and a neutral stimulus followed the mask. When the neutral stimulus appeared, participants were asked to rate the presented photograph on a scale of -3 to 3 based on their immediate preference with -3 indicating “do not like at all”, 0 indicating “neither like nor dislike”, and 3 indicating “like very much.” Images remained on the screen until a response was made. After they finished a set of two tasks, a blank screen was presented for 1,200 ms and then participants were instructed to press the spacebar in order to begin the next trial (see Fig 3). Trials were divided into two blocks. Each block consisted of presentations of 15 preferable stimuli preceding 15 neutral stimuli, 15 less preferable stimuli preceding 15 neutral stimuli, and additionally, 10 filler stimuli containing red dots in the middle of each stimulus preceding 10 filler stimuli without red dots. Each participant was randomly presented with 40 pairs per block. The 15 neutral stimuli which had been paired with preferable prior stimuli in the first block were paired with less preferable prior stimuli in the second block and vice versa. Those 30 pairs of experimental stimuli and 10 pairs of filler stimuli were randomly presented in each block. Preferable/less preferable stimuli were never repeated throughout the experiment.

#### Debriefing

After finishing the experiment, participants were asked to guess the purpose of the experiment and they were debriefed. None of them predicted the purpose correctly and participants reported that they were never aware of this sequence manipulation.

### Results and discussion

Data from filler trials which contained a red dot on a prior stimulus was removed from the analyses. Current neutral stimulus ratings for each participant were normalized to Z scores and mean responses to neutral stimuli were calculated for each participant for the two prior stimulus preference conditions. A repeated-measure ANOVA was conducted on the mean scores with prior stimulus preference as a within-participant factor. According to the results from the analysis, the main effect of prior stimulus preference was significant, *F*(1, 23) = 4.351, *p* = .048, $\eta_{p}^{2}$ = .159. The neutral stimuli were rated more preferably when they followed preferable stimuli (*M* = .0734, *SEM* = 0.0352) than when they followed less preferable stimuli (*M* = —.0734, *SEM* = 0.0352).

The results of Experiment 4 further reinforce the sequential influence on the subsequent preference ratings, ruling out the possibility for a response bias. While participants did not make any explicit manual response for the prior preferable/less preferable stimulus, merely viewing it modulated the subsequent judgment of the subsequent neutral stimulus. The fact that participants were not required to judge preference or make a manual response for the preceding preferable/less preferable stimulus shows that the observed sequence effect was not caused by any manual to keep their response hands near the previously placed location or cognitive bias to continue the previously formed preference. Instead, the results reflect the internal change of preference, which is subject to the sequential modulation.

## General discussion

In a complex social world, we evaluate a variety of stimuli and such evaluations are made in relation to a surrounding context including previously observed stimuli. The present research investigated the existence of preference sequence effects: Does a preference formed on a preceding stimulus influence a subsequently formed preference? Does the preference sequence effect generalize to different types of stimuli and procedures? Does the effect persist after removing response bias? To answer these theoretically important questions, a novel sequential preference rating paradigm was introduced and clear-cut empirical answers to our questions were obtained throughout four experiments. First, we observed a consistent preference sequence effect in rating both artistic photographs and faces: Prior preferable stimuli elicited more preferred responses for subsequent neutral stimuli whereas prior less preferable stimuli triggered less preferred responses for subsequent neutral stimuli in Experiments 1 (artistic photos) and 2 (faces). Also, the sequence effect was generalized to a binary choice task in Experiment 3. Participants made a higher percentage of “prefer” judgments for neutral stimuli when a preferable stimulus preceded them than when a less preferable one did. Second, we disentangled the effect of preferential bias from the effect of response bias: Even when prior stimuli were passively viewed without making any manual response in Experiment 4, a preference sequence effect was obtained.

### Affective bias and sequential effects

Earlier studies on the influence of affective stimuli manipulated either perceptual awareness or contingency awareness in order to study the influence of awareness of affective stimuli on judgments [33]. Stronger priming effects were found when affective stimuli were unconsciously presented than consciously presented, indicating an affective diffuse effect [8, 12, 52–54]. It has been suggested that affective reaction to unconscious stimuli can spill over onto unrelated stimuli without further appraisals, whereas longer exposure durations permit further appraisals, resulting in the dilution of an affective diffuse effects [8, 13].

Even though the present set of experiments are similar to priming experiments in which two stimuli are paired to test the effect of an emotional prime on a neutral target, participants in priming experiments usually did not respond to primes but were only briefly exposed to them. However, participants in the present experiment were presented with seemingly the same type of stimuli and responded to both stimuli, which may have led them to think the two stimuli are independent. This manipulation might have reduced the demand artifacts which were a potential drawback of the previous affective priming research [33]. In fact, when participants were asked to guess the purpose of the experiment or the relationship between the two paired stimuli after the experiment, no one had noticed it.

This manipulation is also qualitatively different from the manipulation of contingency awareness. In the contingency awareness manipulation in which participants are perceptually fully aware of the affective stimulus but they are unaware of its influence on their behavior [30, 55–56], the prime stimulus was an emotional stimulus which is different from the target stimulus. However, in the present study, the preferable/less preferable stimuli were neither representing a positive or negative emotional expression nor being associated with those emotions. A preceding emotional state was formed with a full perceptual awareness of a preceding stimulus which was never associated with affective meaning but evoking preference itself. The obtained findings broaden the affective context effect to the stimulus not associated with affective meaning but evoking preference. The results demonstrated that when people believe two sequentially presented stimuli are not relevant at all, preference for a preceding stimulus assimilates preference for a current stimulus.

Even though further work is needed to examine the role of conscious recognition of the relationship between a previous stimulus and current stimulus in sequential effects, the current finding suggests that when people do not recognize the relevance of a prior stimulus to a current stimulus, preference for a preceding stimulus assimilates preference for a current stimulus. It should be also noted that the influence of a preceding stimulus was effective even when people already finished responding to the preceding stimulus, which is inconsistent with a Zeigarnik effect interpretation.

### Assimilative sequential effects

Previous research regarding the nature of sequential bias has shown contradictory results: Some studies reported a contrast effect [57–59], while other studies reported an assimilation effect [16, 19, 22, 46]. In previous studies, the assimilation effect has been interpreted as a failure to separate an individual stimulus from other stimuli that are simultaneously present, whereas the contrast effect has been interpreted as a result of comparison processes that locate a stimulus within the distribution of successively presented stimuli [58].

In the current study, an assimilation effect was found when two stimuli were successively presented. It is possible that a preceding stimulus might have implicitly influenced the formation of preference for a current stimulus in an assimilative way because the participants did not have to compare a current stimulus to the prior stimulus. In the current study, since participants responded their preferences instead of attractiveness, they did not have to exert their effort for cognitive processing to use the preceding stimulus as a comparison standard. This may have led to an assimilation effect.

The direction of the previous stimulus’s influence has also been suggested to depend on the perceived similarity between the previous and current stimuli [27]. According to this model, participants assimilate their judgments to a given standard if they were made to focus on similarities whereas they contrast their judgments away from the comparison standard if they were made to focus on dissimilarities. Furthermore, similarity was also found to result in an increased perceptual fluency in processing of the current stimulus, leading to more likability for easily processed stimuli [60]. In the present study, a preceding stimulus was seemingly the same type of stimuli with the current stimulus and the only difference was participants’ preference formed toward them. In this situation, participants might have focused on similarity and their judgments may have been subject to an assimilation effect, which is in line with the selective accessibility model.

To our knowledge, it was the first attempt to contrast the sequential influence of preferable and less preferable prior stimulus within the same stimulus in a within-subject design. Even though Damisch et al. (2006) also used pre-rated prime-like stimuli and tested their effects on a neutral stimulus in Study 2, between-subject design was used for each pair and only one pair of stimuli was tested for each participant [20]. The present study extended Damisch et al.’s finding with a design aimed for within-subject tests with relatively large number of stimuli. Kondo et al. (2012) presented face stimuli randomly and found that a face-attractiveness judgment assimilated to the immediately preceding judgment [16]. The present study extended the obtained findings by Kondo et al. by manipulating the sequence of preferable/less preferable stimulus and neutral stimulus based on a preliminary preference rating procedure to compare the sequence effects within the same targets. Even though Kondo et al.’s results were limited to a response trend because they did not control stimulus sequence in terms of preferable state, the present design allowed us to measure sequence effects separately from a response tendency. Particularly in Experiment 4, by adopting a dual task, we could pinpoint a sequential effect without dependence on previous responses.

### Response assimilation

Sequential effects have a potential for response assimilation effects which occurs because participants simply have a tendency to repeat the previous response or a reluctance to move far along the judgment scale. Previous studies also tested this possibility by providing trial-by-trial feedback about the prior judgment [23] or alternating two different tasks [18]. Matthews and Stewart (2009) tested sequential effects in price judgments and found an assimilation effect [23]. In order to remove the possibility of response assimilation, they provided trial-by-trial feedback about the correct value of each product after they had entered their judgment. When people were told the true price of each item after they entered their judgment the assimilation to the preceding judgment largely disappeared and contrast to the preceding item’s true price was replaced by assimilation. Pegors et al. (2015) alternated attractiveness rating and hair darkness rating to measure the mechanisms underlying sequential biases [18]. In Pegors et al.’s study, it has been shown that response bias produced an assimilation effect whereas stimulus bias produced a contrast effect. The present study also directly tested the possibility of response assimilation in Experiment 4. In Experiment 4, participants were exposed to a prime-like stimulus but did not make any manual response for it. However, their preference for a prior stimulus might have been formed unconsciously even though forming a preference was not relevant to their task, and unconsciously formed preference for a prior stimulus might have spilled over onto an unrelated subsequent stimulus or people misattributed their preferences toward a prior stimulus to the subsequent neutral target. However, the obtained assimilation effects without response bias seem to be contradictory with Pegors et al.’s finding in which a contrast effect were found for stimulus bias. However, even though their design adopting a dual task was similar to our Experiment 4, the participants in Pegors et al.’s experiments attended two different values for each task and made manual responses to both different types of rating tasks. In that situation, it is possible that participants adopted different evaluation criteria to rate hair darkness which has a true value and belongs to absolute judgments and to rate attractiveness which does not have a true value and belongs to subjective judgments, resulting in continuously changing psychological contexts. Since a hair darkness rating always alternated with attractiveness rating in Pegors et al.’s experiments, it is hard to tell whether people maintained a criterion for attractiveness during a hair darkness rating. However, in the current study, the participants did not make any manual response but looked at a preferable/less preferable stimulus while they were required to make response only to filler stimuli having a red dot in the middle. The current design allowed a clear demonstration of the source of the sequential effect, maintaining the same evaluation criteria and leaving purely preference-related effects. The obtained findings in Experiment 4 strongly suggest a stimulus-driven sequential bias and there is no evidence for an interpretation of the assimilative sequential bias in terms of a response tendency.

### Theoretical and practical implications

The present inquiry into the presence of the preference sequence effect and consequent empirical findings have some notable theoretical and practical implications. First of all, the present findings add further evidence for a basic processing model of assimilation and contrast effects [61]. Depending on two fundamental types of goals, discrimination and generalization, assimilation or contrast effects are consequent via memory processing, dimensional analyses, or response selection processes. The present study might have elicited a generalization goal affecting a dimensional analysis since the participants rated stimuli based on their own standards applying to all the stimuli they encounter. In other words, the participants did not need to discriminate the presented stimuli into several other categories but they just needed to generally rate how much they liked the presented one. In this situation, as a rule for combining information, a prior stimulus might have functioned as contextual information which is additively combined with target information, resulting in an assimilation effect.

Two conditions should be met for an assimilative sequential bias in preference decisions, (a) previously being exposed to a preference-evoking stimulus in an implicit or explicit way and (b) no need to compare a current stimulus to the prior stimulus. Condition (a) is a prerequisite because it functions as a contextual prime or psychological–emotional in particular—context. However, it does not mean that the preceding stimulus should be an emotional [8] or affect-laden stimulus [33]. It could be any newly encountered preference-evoking stimulus, since preference is based on hedonic value. This is particularly the case for such a stimulus as art piece [62]. Condition (b) could be met being unconscious of a prime stimulus itself as previous affective priming studies revealed [12], or unaware of the relationship between the preceding stimulus and a current stimulus as the present research demonstrated. Response bias or completeness of a preceding stimulus is not the conditions which need to be met to produce a preference sequence effect. The current study broadens the scope of sequential influence on preference decisions by adding new empirical evidence to extant findings and theories on assimilative sequential biases. Future research should be more cautious when presenting a stimulus set sequentially and examining preference.

The present demonstration of the preference sequence effect is also of practical importance. In real world situations such as advertisements, displayed artworks, and grading essays [22], people evaluate the same type of stimuli that are sequentially presented. Also, in experimental situations, such as during preference judgment tasks, stimuli from the same category are often presented sequentially. So far, sequential effects were examined in real settings such as performance in Olympic games [21–22], performance evaluation in Idol television series [63], price judgments in market place [23], essay evaluation [22], and attractive ratings in online dating situation [19], as well as in experimental settings by randomly presenting to-be-rated stimuli [38] or inserting an emotional face stimulus prior to a to-be-rated stimuli [8]. The current study which was conducted in a laboratory-controlled setting provides a bridge to the findings from field studies. The novel experimental paradigm revealed the existence of the preference sequence effect even in the absence of response bias, providing practical and ecological implications for sequential evaluation situations both in real world and experiment settings.

## Conclusion

The central finding of the present study was that preference for a current stimulus differed depending on preference for a prior stimulus in a trial-by-trial manner. The present study has theoretical and practical implications by providing further evidence for conditions eliciting assimilative sequential bias in preference decisions. The results highlight the need for a consideration of trial sequence as a factor creating a psychological context and will add to a greater understanding of how sequential context influences affective evaluations.

## Supporting information

S1 File Dataset of experiments. (ZIP)
